# Sex-related differences of invasive therapy in patients with aneurysmal subarachnoid hemorrhage

**DOI:** 10.1007/s00701-022-05345-0

**Published:** 2022-08-19

**Authors:** S. Y. Bögli, D. Utebay, N. Smits, L. P. Westphal, L. Hirsbrunner, S. Unseld, E. Keller, G. Brandi

**Affiliations:** 1 Institute for Intensive Care Medicine, University Hospital Zurich, Zurich, Switzerland; 2 Department of Neurology, University Hospital Zurich, Zurich, Switzerland; 3 Department of Neurosurgery and Clinical Neuroscience Center, University Hospital and University of Zurich, Zurich, Switzerland

**Keywords:** Aneurysmal subarachnoid hemorrhage, Gender medicine, Stroke, Intensive care

## Abstract

**Background:**

Sex-related differences in patients with aneurysmal subarachnoid hemorrhage (aSAH) exist. More females than males are affected. Aneurysm location is associated to sex. The relationship between sex and outcome, however, is unclear. Possible differences in management might influence the occurrence of primary and secondary brain injury and thus outcome. The study compares demographics, intensity of treatment, complications, and outcome among females and males with aSAH.

**Methods:**

All consecutive patients with aSAH admitted to the neurocritical care unit, University Hospital Zurich over a 5-year period were eligible in this retrospective study. Patients’ characteristics, comorbidities, aSAH severity, frequency of vasospasm/delayed cerebral ischemia, frequency of invasive interventions, and 3-month outcome were compared by sex. Univariate analysis was performed with the data dichotomized by sex, and outcome. Multivariate analysis for prediction of outcomes was performed.

**Results:**

Three hundred forty-eight patients were enrolled (64% females). Women were older than men. Comorbidities, scores at admission, and treatment modality were comparable among males and females. Vasospasm and DCI occurred similarly among females and males. Interventions and frequency of intraarterial spasmolysis were comparable between sexes. In the multivariate analysis, increasing age, female sex, increasing comorbidities, WFNS and Fisher grade, and presence of delayed cerebral ischemia were predictors of unfavorable outcome when considering all patients. However, after excluding death as a possible outcome, sex did not remain a predictor of unfavorable outcome.

**Conclusions:**

In the study population, women with aSAH might have present a worse outcome at 3 months. However, no differences by sex that might explain this difference were found in intensity of treatment and management.

## Introduction

Sex-related differences in patients with aneurysmal subarachnoid hemorrhage (aSAH) exist [13]. Generally, females are more likely to suffer from aSAH [10, 24, 28, 30, 35]. Possible reasons discussed are the intrinsic weakness of the vessel walls, collagen, and elastin interference factors, as well as hormonal influence that may play a role in aneurysm formation in women [17, 31]. Furthermore, some risk factors (e.g., smoking) increase the odds of aneurysm rupture to a greater extent in women [12, 20, 21]. Aneurysm location itself also appears to be associated to sex. Most aneurysms in females are located along the internal carotid artery while in males, they are located along the anterior cerebral artery [1, 16]. Hemodynamic factors in relation to sex-specific cerebral vascular anatomy might be the reason for these sex-related differences in aneurysm location [22]. The relationship between sex and vasospasm as well as delayed cerebral ischemia (DCI), however, is poorly characterized. The available data on sex hormones and vasospasm/DCI are contradicting [4, 9, 15, 34]. Similarly, data regarding the effect of sex and gender on clinical course, intensity of treatment, and outcome are controversial [3, 11, 23]. Potential sex-related differences in patients with aSAH might arise during the hospital course. These differences might have an impact on disease progression. We previously reported that male patients with spontaneous intracerebral hemorrhage were almost three times as likely to receive an external ventricular drain (EVD) in comparison to women in the absence of radiographic or clinical differences. We suggested that a form of “gender-bias” might have influenced the decision-making process for the insertion of an EVD [33]

The identification of sex- and gender-related differences in patients with aSAH is of great interest to evaluate the current monitoring and treatment strategies in order to offer a more personalized management and to better distribute the available resources. The aim of this study was to compare clinical characteristics, management, intensity of treatment, and outcome among males and females with aSAH.

## Methods

### Patient population, inclusion and exclusion criteria

We retrospectively reviewed the medical records of all consecutive patients with aSAH admitted to the neurocritical care unit (NCCU) of the University Hospital Zurich between January 2016 and December 2021. The only patients included were adults (≥ 18 years old) with aSAH (i.e., with imaging evidence of a ruptured aneurysm). Exclusion criteria were (1) patients with only unruptured, traumatic, fusiform, dissecting, or mycotic aneurysms; (2) patient’s written or documented oral refusal to have their data analyzed for research projects. The local ethic committee approved the study. It was performed in accordance with the ethical standards as laid down in the 2013 Declaration of Helsinki. STROBE guidelines were used to draft the manuscript.

### Patients’ management

#### Aneurysm securing

Based on our institutional protocol, aneurysm repair occurs within 24 h from bleeding by surgical or endovascular means. The decision for surgical or endovascular treatment is taken by the neurosurgeons and the neuroradiologist in charge.

#### External ventricular drain (EVD; BACTISEAL® EVD Catheter, CODMAN, Johnson & Johnson, Raynham, MA, USA)

The decision for the insertion of an EVD is taken by the treating neurosurgeon (at times in consultation with the neuroradiologists) using the following criteria: (1) In case of enlargement of the third ventricle and of the temporal horns of the lateral ventricles (i.e., ventriculomegaly with signs of acute occlusive hydrocephalus), (2) in case direct monitoring of the intracranial pressure (ICP) is essential such as in unconscious to comatose patients. In these patients, EVD is inserted within the first hours after admission.

#### Invasive multimodal neuromonitoring

In case of impaired consciousness either due to the severity of disease itself or due to the need for deep sedation (e.g., issues with ICP or artificial ventilation), beyond ICP-monitoring, probes for multimodal neuromonitoring before the vasospasm phase are inserted mostly bilateral into the frontal lobes. The invasive multimodal neuromonitoring includes hourly evaluated cerebral microdialysis (evaluating glucose, lactate and pyruvate levels; CMA 70, CMA Mikrodialysis, Solna, Sweden), continuous measurement of brain tissue oxygenation, and depending on the depth of sedation or previous seizures/seizure risks continuous electroencephalography. The decision for the insertion of invasive multimodal neuromonitoring is taken by the neurocritical care specialists and the treating neurosurgeons.

During the vasospasm phase (at least until day 14 after aSAH), all patients (irrespective of initial clinical or radiologic grade) are treated at the NCCU. For earliest possible detection of delayed cerebral ischemia (DCI) and vasospasm, serial assessment of neurological function (up to hourly depending on clinical state) and daily transcranial Doppler measurements are performed. Nimodipine is administered to all patients either orally or intravenously for the prevention of vasospasm using standardized dosages. In patients with either clinical deterioration or worsening of invasive neuromonitoring values, CT scans (including angiography and perfusion imaging) are performed (available at all time).

#### Intraarterial spasmolysis

In case of symptomatic vasospasm confirmed by CT-angiography with corresponding clinical deterioration or corresponding perfusion deficit upon perfusion imaging that are refractory to hemodynamic therapy, intraarterial spasmolysis using nimodipine is performed (available during the whole week 24 h per day). The decision for the performance of an intraarterial spasmolysis is taken inter-disciplinary by the neurocritical care specialist, the neuroradiologist, and the neurosurgeon.

### Data collection

Data collection was performed by scanning the electronic health records for demographic characteristics, clinical and radiological information (Fisher grade, location of ruptured aneurysm, presence of intracerebral as well as intraventricular or subdural hemorrhage including location, and presence of ventriculomegaly, presence of compressed basal cisterns), treatment modality (clipping, coiling or flow-diverter based therapy), clinical course (occurrence of ventriculostomy-related infection, vasospasm, or DCI), and outcome data. The radiological findings aside from DCI as well as vasospasm were extracted from initial CT (including CT-angiography) imaging. DCI was defined as a cerebral infarction on CT or MRI excluding other causes (such as endovascular treatment or clipping) not on the CT scan 24–48 h after aneurysm occlusion [19], excluding the clinical correlate of DCI (occurrence of focal neurological impairment or decrease of 2 or more Glasgow Coma Scale (GCS) points for at least 1 h) to reduce the bias caused by the retrospective design of this study. The diagnosis of vasospasm was only used for cases with respective description on CT, MR, or digital subtraction angiography. The Charlson Comorbidity Index (CCI) was assessed to evaluate relevant comorbidities [7]. Outcome is presented using the Glasgow Outcome Scale Extended (GOSE) extracted from routine follow-up consultations at 3 months (which include a neurological examination, as well as a description of current occupation including the percentage of working capability). A dichotomized GOSE in favorable (GOSE 5–8) and unfavorable (GOSE 1 to 4) was considered in the analysis, as in previous studies [5, 32]. Furthermore, we analyzed separately patients who died (GOSE 1) and survivors. These were further dichotomized in patients with GOSE 2–4 (survivors with unfavorable outcomes) versus patients with excellent outcome (GOSE 7–8).

### Statistical analysis

Statistical analysis was performed using SPSS version 26. Data was always dichotomized by sex (male vs. female) or depending on outcome. Descriptive statistics are reported as counts/percentages, mean ± standard deviation, or as median including the interquartile range as appropriate. All continuous data were tested for normality using Shapiro–Wilk’s test. Categorical variables are compared with Pearson’s χ2 or Fisher’s exact test, continuous/ordinal variables using Student’s *t*-tests or Mann–Whitney *U* tests for parametric and non-parametric data, respectively, where appropriate.

Univariate logistic regression permitted to find predictive variables associated with either outcome group: unfavorable outcome (GOSE 1 to 4), or death (GOSE 1), survivors with unfavorable outcomes (GOSE 2–4) or excellent outcome (GOSE 7–8) by a *P* value < 0.05 and entered in multivariable logistic regression models. Due to collinearity of the specific imaging features (i.e., intraventricular hemorrhage, ventriculomegaly etc.) and the Fisher grade, they were left out of the multivariate model. Furthermore, we excluded the interventions from the multivariate analysis as these are performed depending on clear criteria and thus inadequate for outcome prediction. The odds ratios (ORs) were calculated and expressed with the corresponding 95% confidence intervals (95% CI). All tests were performed two-sided and a *P* value ˂0.05 was considered statistical significant.

## Results

Overall, 348 patients with SAH met the inclusion criteria. The majority of patients were female (*N* = 222, 64%). Patients’ demographics, comorbidities, and severity of aSAH at admission are presented in Table 1. Women were older than men (*p* = 0.001)*.* No differences in presence of comorbidities, as well as scores at admission, were found among females and males. More men than women had an aneurysm of the anterior communicating artery (*p* = 0.0002), while more women than men had an aneurysm of the posterior communicating artery (*p* = 0.017) (Table 1). Radiological findings, including presence of intracerebral hemorrhage, intraventricular hemorrhage, subdural hematoma, ventriculomegaly, and blood in the basal cisterns, were comparable among men and women, as shown in Table 2.

**Table 1:** Demographics, scores at admission, and aneurysm location

		All (N)	Male (N)	Female (N)	*p* value
		348	126 (36.2)	222 (63.8)	< 0.001
*Age (years)*		57 ± 13.2	54 ± 11.6	59 ± 13.8	0.001
*CCI*	Total	0 [0,2]	0 [0,2]	0 [0,2]	0.853
	aMI	24 (6.9)	11 (8,7)	13 (5.9)	0.379
	CHF	19 (5.5)	7 (5.6)	12 (5.4)	1
	PVD	19 (5.5)	3 (2.4)	16 (7.2)	0.083
	CVI	15 (4.3)	3 (2.4)	12 (5.4)	0.272
	Dementia	2 (0.6)	0 (0.0)	2 (0.9)	0.537
	CLD	36 (10.3)	17 (13.5)	19 (8.6)	0.199
	RD	22 (6.3)	4 (3.2)	18 (8.1)	0.106
	PU	8 (2.3)	4 (3.2)	4 (1.8)	0.468
	mLD	14 (4.0)	7 (5.6)	7 (3.2)	0.395
	m/sLD	4 (1.1)	2 (1.6)	2 (0.9)	0.623
	m/mD	17 (4.9)	8 (6.3)	9 (4.1)	0.438
	Diabetes	2 (0.2)	1 (0.6)	1 (0.5)	1
	Hemiplegia/paraplegia	7 (2.0)	4 (3.2)	3 (1.4)	0.636
	KD	20 (5.7)	9 (7.1)	11 (5.0)	0.665
	Malignant tumors	25 (7.2)	10 (7.9)	15 (6.8)	0.924
	Solid metastatic tumor	4 (1.1)	0 (0.0)	4 (1.8)	0.301
	AIDS	1 (0.3)	1 (0.8)	0 (0.0)	0.362
*WFNS*	1	117 (33.7)	38 (30.2)	79 (35.7)	0.375
	2	71 (20.5)	28 (22.2)	43 (19.5)	
	3	14 (4.0)	3 (2.4)	11 (5.0)	
	4	68 (19.6)	28 (22.2)	40 (18.1)	
	5	77 (22.2)	29 (23.0)	48 (21.7)	
*Fisher*	1	13 (3.8)	4 (3.2)	9 (4.1)	0.819
	2	24 (6.9)	8 (6.3)	16 (7.3)	
	3	144 (41.6)	56 (44.4)	88 (40.0)	
	4	165 (47.7)	58 (46)	107 (48.6)	
*Aneurysm location*	Anterior	276 (79.3)	103 (81.7)	173 (77.9)	0.413
	ICA	27 (7.8)	9 (7.1)	18 (8.1)	0.908
	MCA	88 (25.3)	28 (22.2)	60 (27.0)	0.388
	AComA	108 (31.0)	55 (43.7)	53 (23.9)	< 0.001
	ACA	6 (1.7)	1 (0.8)	5 (2.3)	0.424
	VA	11 (3.2)	5 (4.0)	6 (2.7)	0.5357
	PCA	4 (1.1)	1 (0.8)	3 (1.4)	1
	PICA	18 (5.2)	5 (4.0)	13 (5.9)	0.615
	PComA	53 (15.2)	11 (8.7)	42 (18.9)	0.017
	Basilar	18 (5.2)	6 (4.8)	12 (5.4)	0.993

*CCI* Charlson Comorbidity Index, *aMI* acute myocardial infarction, *CHF* congestive heart failure, *PVD* peripheral vascular disease, *CVI* cerebral vascular insult, *CLD* chronic lung disease, *RD* rheumatic disease, *PU* peptic ulcer, *mLD* mild liver disease, *m/sLD* moderate to serious liver disease*, m/mD* mild to moderate diabetes, *Diabetes* diabetes with chronic complications, *KD* kidney disease, *WFNS* world federation of neurosurgery, *Fisher* Fisher grade, *ICA* internal carotid artery, *MCA* middle cerebral artery, *AComA* anterior communicating artery, *ACA* anterior cerebral artery, *VA* vertebral artery, *PCA* posterior cerebral artery, *PICA* posterior inferior cerebellar artery, *PComA* posterior communicating artery, *Basilar* basilar artery. Data are expressed as number (percentage), mean ± standard deviation, or median [interquartile range]

**Table 2:** Radiological features, intensity of treatment, clinical course, and outcomes

		All (N)	Male (N)	Female (N)	*p* value
		348	126 (36.2)	222 (63.8)	< 0.001
Radiological features	ICH	99 (28,4)	38 (30.2)	61 (27.5)	0.622
	SDH	33 (9,5)	17 (13.5)	16 (7.2)	0.054
	Ventriculomegaly	182 (52.3)	66 (52.4)	116 (52.3)	1
	Basal cisterns	221 (63.5)	82 (65.9)	138 (62.2)	0.563
	IVH	254 (73.0)	88 (69.8)	157 (70.7)	0.903
Clinical course					
* Aneurysm repair*	Clipping	161 (46.3)	52 (41.3)	109 (49.1)	0.172
	Coiling	160 (46.0)	60 (47.6)	100 (45.0)	
	Flow-diverter	10 (2.9)	6 (4.8)	4 (1.8)	
	Trapping	4 (1.1)	3 (2.4)	1 (0.5)	
* EVD*		181 (52.0)	69 (54.8)	112 (50.5)	0.503
* Multimodal neuromonitoring*		57 (16.4)	24 (19.0)	33 (14.9)	0.366
* LD*		23 (6.6)	5 (4.0)	18 (8.1)	0.178
* VRI*		51 (14.7)	19 (15.1)	32 (14.4)	0.876
* Re-rupture*		2 (0.6)	1 (0.8)	1 (0.5)	1
* Vasospasm*		209 (60.1)	75 (59.5)	134 (60.4)	0.91
* DCI*		93 (26.7)	37 (29.4)	56 (25.5)	0.45
* Intraarterial spasmolysis*		64 (18.4)	20 (15.9)	44 (19.8)	0.391
Outcome					
* ICU-LOS (days)*		19 ± 13.2	18 ± 11.8	19 ± 27.5	0.579
* GOSE*					0.005
	1	61 (17.6)	17 (13.6)	44 (19.9)	
	2	11 (3.2)	2 (1.6)	9 (4.1)	
	3	55 (15.9)	22 (17.6)	33 (14.9)	
	4	36 (10.4)	8 (6.4)	28 (12.7)	
	5	45 (13.0)	22 (17.6)	23 (10.4)	
	6	42 (12.2)	23 (18.4)	19 (8.6)	
	7	54 (15.6)	13 (10.4)	41 (18.6)	
	8	42 (12.1)	18 (14.4)	24 (10.9)	
* Unfavorable outcome*	GOSE 1–4	163 (47.1)	49 (39.2)	114 (51.6)	0.013
* WLST*		40 (13.8)	11 (8.7)	29 (13.1)	0.294
* Immediate palliation*		11 (3.2)	3 (2.4)	8 (3.6)	0.752

*ICH* intracerebral hemorrhage, *SDH* subdural hemorrhage, *basal cisterna* presence of blood in the basal cisterna, *IVH* intraventricular hemorrhage, *EVD* extern ventricular drain insertion, *LD* lumbar drain insertion, *VRI* ventriculostomy-related infection, *DCI* delayed cerebral infarct, *ICU-LOS* length of stay at the intensive care unit, *GOSE* Glasgow outcome scale extended, *WLST* withdrawal of life-sustaining therapies. Data are expressed as number (percentage), mean ± standard deviation, or median [interquartile range]

No differences by sex in treatment modality to aneurysm repair were found, as presented in Table 2, as well as in frequency of insertion of EVD, lumbar drain, and multimodal neuromonitoring. Vasospasm occurred similarly among men and women, as well as DCI, as shown in Table 2. An intraarterial spasmolysis was performed similarly among men and women, as shown in Table 2.

**Fig. 1:**
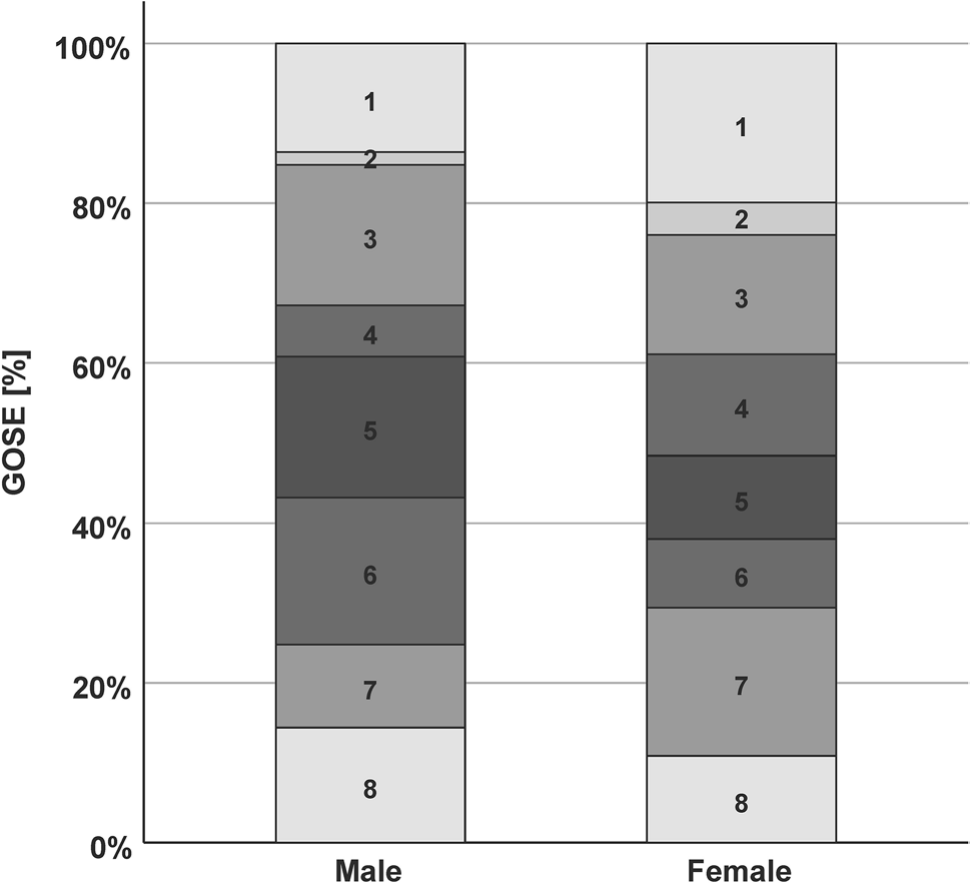
Data on Glasgow Outcome Scale extended at 3 months for females and males. Data are expressed as percentage (%)

Length of ICU-stay was similar among men and women, as reported in Table 2. Data on GOSE at 3 months are presented in Table 2 and Fig. 1. An unfavorable outcome was more frequently in females than males (50.5 vs. 36.5%, *p* = 0.013). The findings were similar after stratification of the patients by severity based on the Fisher scale (grade 1–2 vs. 3–4), and after stratification by clinical neurological presentation based on WFNS grading system (WFNS 1–3 vs. WFNS 4–5) (data not shown).

Frequency of withdrawal of life-sustaining therapies as well as of immediate palliation was comparable among women and men, as shown in Table 2.

Based on the results of the univariate analysis evaluating factors associated with unfavorable outcome (GOSE 1–4), death (GOSE1), survivors with unfavorable outcome (GOSE 2–4), and excellent outcome (GOSE 7 and 8), multivariate analysis was performed (Table 3). Increasing age, female sex, increasing CCI, increasing WFNS, and Fisher, as well as presence of DCI, were independently associated with unfavorable outcome. When considering only survivors, sex lost its predictive value. Increasing age, increasing WFNS, and Fisher scores were predictors of death. Lower Charlson Comorbidity index and WFNS score were predictors of excellent outcomes.

**Table 3:** Univariate and multivariate outcome prediction

For the prediction of:	Univariate analysis 1 GOSE 1–4		Multivariate analysis 1 GOSE 1–4		Univariate analysis 2 GOSE 2–4		Multivariate analysis 2 GOSE 2–4		Univariate analysis 3 GOSE 1		Multivariate analysis 3 GOSE 1		Univariate analysis 4 GOSE 7–8		Multivariate analysis 4 GOSE 7–8	
	OR (95%-CI)	*p* value	OR (95%-CI)	*p* value	OR (95%-CI)	*p* value	OR (95%-CI)	*p* value	OR (95%-CI)	*p* value	OR (95%-CI)	*p* value	OR (95%-CI)	*p* value	OR (95%-CI)	*p* value
*Age (years)*	1.05 (1.03–1.07)	< 0.001	1.04 (1.02–1.06)	< 0.001	1.03 (1.01–1.05)	0.011	1.02 (1.00–1.05)	0.047	1.09 (1.06–1.13)	< 0.001	1.10 (1.07–1.14)	< 0.001	0.98 (0.97–1.00)	0.086		
*Sex (female)*	1.65 (1.06–2.58)	0.027	1.99 (1.14–3.49)	0.016	1.55 (0.93–2.59)	0.091			1.58 (0.86–2.90)	0.141			1.26 (0.77–2.08)	0.358		
*CCI (total)*	1.40 (1.18–1.66)	< 0.001	1.24 (1.03–1.50)	0.027	1.27 (1.07–1.51)	0.007	1.17 (0.98–1.41)	0.114	1.30 (1.11–1.52)	0.001	1.06 0.88–1.30	0.532	0.75 (0.61–0.93)	0.007	0.79 (0.64–0.99)	0.036
*WFNS (unit)*	1.83 (1.57–2.13)	< 0.001	1.62 (1.35–1.94)	< 0.001	1.65 (1.39–1.95)	< 0.001	1.45 (1.12–1.75)	< 0.001	1.88 (1.53–2.32)	< 0.001	1.88 (1.45–2.43)	< 0.001	0.56 (0.49–0.70)	< 0.001	0.65 (0.54–0.80)	< 0.001
*Fisher (unit)*	3.89 (2.63–5.74)	< 0.001	2.50 (1.60–3.92)	< 0.001	2.91 (1.93–4.39)	< 0.001	2.10 (1.34–3.30)	0.001	5.21 (2.74–9.87)	< 0.001	3.30 (1.58–6.88)	0.001	0.49 (0.36–0.66)	< 0.001	0.71 (0.50–1.02)	0.060
*Aneurysm location (anterior as reference)*	0.93 (0.55–1.56)	0.771			1.18 (0.63–2.21)	0.598			1.06 (0.98–1.15)	0.133			1.60 (0.85–2.98)	0.143		
*Presence of ICH*	3.96 (2.38–6.59)	< 0.001			4.04 (2.30–7.08)	< 0.001			2.09 (1.18–3.73)	0.012			0.25 (0.13–0.50)	< 0.001		
*Presence of SDH*	4.77 (2.01–11.32)	< 0.001			4.34 (1.71–11.02)	0.002			2.63 (1.20–5.76)	0.016			0.15 (0.04–0.64)	0.010		
*Ventriculomegaly*	3.45 (2.21–5.38)	< 0.001			2.96 (179–4.90)	< 0.001			3.08 (1.66–5.71)	< 0.001			0.28 (0.17–0.46)	< 0.001		
*Presence of compressed basal cisterns*	2.22 (1.41–3.49)	0.001			1.79 (1.08–2.99)	0.025			2.74 (1.40–5.38)	0.003			0.43 (0.27–0.70)	0.001		
*Presence of IVH*	5.03 (2.94–8.59)	< 0.001			3.22 (1.82–5.69)	< 0.001			16.03 (3.84–67.00)	< 0.001			0.41 (0.25–0.67)	< 0.001		
*Aneurysm repair modality (coiling as reference)*	1.19 (0.91–1.57)	0.208			1.21 (0.98–1.65)	0.220			1.075 (0.76–1.52)	0.681			1.00 (0.74–1.35)	0.989		
*Presence of EVD*	4.96 (3.14–7.85)	< 0.001			4.27 (2.54–7.17)	< 0.001			3.89 (2.05–7.38)	< 0.001			0.24 (0.14–0.40)	< 0.001		
*Presence of multimodal neuromonitoring*	6.80 (3.3–14.02)	< 0.001			9.04 (4.24–19.27)	< 0.001			1.17 (0.57–2.43)	0.666			0.08 (0.02–0.32)	< 0.001		
*Presence of LD*	0.37 (0.14–0.97)	0.042			0.40 (0.13–1.21)	0.104			0.43 (0.10–1.86)	0.256			1.76 (0.74–4.22)	0.203		
*Presence of VRI*	0.95 (0.52–1.73)	0.870			1.24 (0.65–2.38)	0.521			0.48 (0.18–1.25)	0.133			1.27 (0.67–2.43)	0.468		
*Presence of vasospasm*	1.24 (0.81–1.91)	0.325			2.41 (1.40–4.16)	0.001			0.36 (0.21–0.64)	< 0.001			0.61 (0.38–0.98)	0.04		
*Presence of DCI*	1.68 (1.04–2.71)	0.036	1.80 (1.01–3.20)	0.047	2.21 (1.30–3.77)	0.003	1.93 (1.07–3.45)	0.028	0.71 (0.36–1.37)	0.306			0.50 (0.28–0.91)	0.022	0.56 (0.30–1.06)	0.073
*Need of Intraarterial spasmolysis*	1.20 (0.69–2.07)	0.517			1.95 (1.09–3.47)	0.024			0.16 (0.06–0.64)	0.007			0.95 (0.52–1.76)	0.881		

Univariate and multivariate logistic regression models for the prediction of unfavorable outcome (GOSE 1–4, model 1), unfavorable outcome except death (GOSE 2–4, model 2), death (model 3), and of excellent outcome (GOSE 7 and 8, model 4). Values are indicated as odds ratio (95% confidence interval, CI confidence interval). *GOSE* Glasgow outcome scale extended, *CCI* Charlson Comorbidity Index, *WFNS* World Federation of Neurosurgery, *ICH* intracerebral hemorrhage, *SDH* subdural hemorrhage, *IVH* intraventricular hemorrhage, *EVD* extern ventricular drain, *LD* lumbal drain, *DCI* delayed cerebral infarction

## Discussion

This study investigates the role of sex on management in patients with aSAH. The strength of this study lies in the use of a database of consecutive patients with aSAH during a 5-year period with several parameters collected and no missing data. Similarly to previous findings, in the study population, more females than males were affected by aSAH [11, 27, 30]. Several reasons could explain this female prevalence, including hormonal influences on vascular remodeling and potentially increased frequency of vessel wall weakness and risk of aneurysmal rupture in females [13]. With regard to aneurysm securing strategies, management, and frequency of complications, no differences by sex were found in the study population.

Previous studies, particularly in the field of cardiology, reported that women are less likely to receive evidence-based acute treatments for acute coronary syndrome than men [6, 18]. A form of gender bias — mostly unconscious — was the presumed reason for less aggressive treatment for women. Similarly, our group recently reported that at our institution women with spontaneous intracerebral hemorrhage are less likely than men to receive an EVD despite comparable frequency of hydrocephalus/ventriculomegaly. Multivariate analysis proved male gender to be independently associated with EVD insertion in these patients even after correction for location and severity of bleeding [33].

At our NCCU, patients with aSAH are treated based on a standardized protocol, which includes early aneurysm securing, ICP-monitoring in case of severely decreased vigilance, EVD insertion in case of ventriculomegaly or clinical signs for increased ICP, as well as serial TCCD measurements and insertion of multimodal neuromonitoring for the early detection of symptomatic vasospasm and DCI. Decisions are taken in multidisciplinary consensus between neurosurgeons, neuroradiologists, and neurocritical care specialists. This protocol based, inter-disciplinary approach may minimize the influence of “gender bias”[26].

With regard to outcome, previous findings in patients with aSAH are inconclusive [13]. In our study population, women fared worse than men did. We suppose that this result might be partly due to the older age of the females. In fact it is well-known that older age is a determinant for poor outcome in patients with aSAH [8, 25]. In the multivariate analysis, increasing age is independently associated with unfavorable outcome. Furthermore, female sex per se was a predictor of unfavorable outcome if including death as a possible outcome, confirming the recent preliminary data from a large cohort (*n* > 8000 patients) of aSAH patients [2]. This finding is quite interesting and it might also be secondary to the treatment targets that we set without taking into account patients’ sex. For instance, the blood pressure values targeted are different in presence of vasospasm. In these cases, controlled arterial hypertension is induced to maintain/reach sufficient cerebral blood flow stable to prevent or treat DCI. However, induced hypertension might lead to serious adverse events, as cardiac arrhythmia, myocardial infarction, pulmonary edema, brain edema, hemorrhagic infarction, and rebleeding [14]. Because sex-related differences in cardiovascular conditions exist [29], these should be considered in the management of patients with SAH. Previous studies on clinical outcomes following aSAH and sex are not conclusive [13], probably also due to methodological issues on outcome assessment, which complicate comparison of findings across studies. To improve understanding of the role sex plays in this context, it is imperative that outcomes studies include sex as a predictor variable in their analyses and examine sex-specific effects of interventions.

The findings that increasing age, increasing WFNS, and Fisher scores are predictors of death after aSAH are not new. Interestingly, despite a trend for more excellent outcome in females than males, this finding was not confirmed in the multivariate analysis.

### Limitations

The study has some limitations. Firstly, this is the experience of a single-center, limiting the generalizability of our findings. Secondly, due to the retrospective nature of the study, we cannot exclude detection and referral biases. Thirdly, despite the large number of parameters collected, some outcome of interest as frequency of cardiovascular complications during the NCCU-stay are not available, permitting only speculations on the reported differences in outcome by sex. Fourthly, we can only make some speculations on the reasons of the worse outcome for females compared to males. Some well-known determinants of poor outcomes (for instance, aneurysmal re-rupture, diffuse brain swelling on CT scan, and fever) in patients with aSAH were not taken into account in the analysis. Finally, outcome was assessed only 3 months following the SAH, limiting the evaluation of a possible neurological improvement later on.

## Conclusion

No differences in the frequency of invasive therapies between the sexes could be found in this study. While this study did not directly evaluate the effect of using a protocol, the results, particularly if considering the previous published findings referring to patients with spontaneous intracerebral hemorrhage, suggest that a well-established inter-disciplinary decision-making process might minimize sex-related bias in treatment. The results also implicate that beyond age, there might be more subtle differences or even treatment-related complications that might have caused the difference in outcome that will have to be taken into account in future prospective studies.
